# Evaluation of the respiratory function in argentinian young adults without respiratory pathology from rosario's city

**DOI:** 10.1186/1939-4551-8-S1-A113

**Published:** 2015-04-08

**Authors:** Ledit Ardusso, Mariela Formaggio, Luciano Rovetto, Matias Duarte, Guillermo Mujica, Nora Figueroa, Veronica Estrella, Rut Aguero, Jorge Molinas

**Affiliations:** 1 National University of Rosario, Argentina

## Background

Have not been found to date, for young adults in our environment, spirometric predictive values depending on the age, sex and height, and it would be of interest his determination for the construction of a table of normality for this group, since this actually requires the use of values from foreign populations

## Methods

Were studied 232 students (151 women and 81 men) with ages understood between 18 and 29 years (x=21,68±2,24) who were in the first three years of the School of Medicine at the Faculty Medical Sciences of Rosario's National University, Argentina during the year 2014. The sample was randomly selected and students gave their informed consent to undergo spirometry effort (with spirometer "Multispiro III") based technique ATS, registering the values of forced vital capacity (FVC) and forced expiratory volume in one second (FEV1). EPI INFO program was used for the statistical treatment of the data and the results was expressed in tables based on age groups and four groups according to quartiles of stature by sex.

## Results

It was found a mean FVC of 4.05±1.00 liters and a mean FEV1 of 3.70±0.84 liters.

In table 1 can be observed the average FVC and FEV1 according to sex, age, and height quartiles by sex.

**Table 1:** 

	Age (years) / Height (m)	18/19	20/21	22/23	24/25	26/27	28/29
**FVC men (liters)**	**<1.71**	5,27±0,28	4,75±0,70	4,64±0,51	4,89±0,23	5,19±0	3,88±0
	
	**1.72 – 1.75**	-	4,77±0,44	4,61±0,48	4,75±0	-	5,82±0
	
	**1.76-1.80**	5,55±1,24	5,08±0,70	5,24±0,59	4,85±0,76	5,47±0	4,70±1,09
	
	**>1.80**	4,98±0,07	6,01±0,80	6,05±0	5 ,61±0,62	6,66±0	-

**FEV1 men (liters)**	**<1.71**	4,78±0,58	4,35±0,49	4,04±0,32	4,52±0,25	4,45±0	3,74±0
	
	**1.72 – 1.75**	-	4,35±0,38	4,26±0,42	3,97±0	-	5,22±0
	
	**1.76-1.80**	5,35±1,20	4,74±0,66	4,70±0,57	4,14±0,80	4,92±0	4,12±0,35
	
	**>1.80**	4,67±0,02	5,51±0,85	5,08±0	4,93±0,86	6,01±0	-

**FVC women (liters)**	**<1.60**	3,52±0,90	3,28±0,55	2,93±0,51	3,21±0,64	3,02±0,43	3,48±0
	
	**1.60 – 1.62**	3,07±0,36	3,24±0,48	3,31±0,30	3,49±0,48	3,00±0	-
	
	**1.63-1.67**	3,34±0,09	3,57±0,45	4,06±0,83	3,47±0,46	3,42±0	3,38±0,09
	
	**>1.67**	3,64±0,79	4,03±0,58	3,83±0,52	4,14±0	3,45±0,58	-

**FEV1 women (liters)**	**<1.60**	3,32±0,81	2,96±0,40	2,78±0,42	2,49±0,20	2,59±0,35	3,19±0
	
	**1.60 – 1.62**	3,11±0,42	2,90±0,28	3,01±0,37	3,53±0,14	2,99±0	-
	
	**1.63-1.67**	3,09±0,10	3,32±0,42	3,68±0,74	3,15±0,23	3,34±0	2,86±0,10
	
	**>1.67**	3,54±0,57	3,70±0,53	3,49±0,47	3,60±0	3,20±0,54	-

## Conclusions

This paper presents, in young adults, normal values of FVC and FEV1 in a sample of healthy individuals from the general population of the city of Rosario, Argentina and is expected to be useful for the construction of equations for predictive values.

